# Cytotoxic Effects of Sorafenib, Lapatinib, and Bevacizumab, Alone and in Combination, on Medullary Thyroid Carcinoma Cells

**DOI:** 10.3390/curroncol32110607

**Published:** 2025-10-31

**Authors:** Gülşah Altun, Özlem Yönem

**Affiliations:** 1 Department of Internal Medicine, Sivas Numune State Hospital, 58060 Sivas, Türkiye; 2 Department of Gastroenterology, Bodrum Memorial Hospital, 48400 Bodrum, Türkiye; ozlem.yonem@memorial.com.tr

**Keywords:** medullary thyroid carcinoma, Sorafenib, Lapatinib, Bevacizumab, targeted therapy, combination therapy, RET mutation

## Abstract

**Simple Summary:**

Thyroid cancer is one of the most common endocrine cancers, and medullary thyroid carcinoma is a rare but aggressive type that can be difficult to treat. Surgery is often the first option, but many patients eventually need drug treatments. Current medicines, called tyrosine kinase inhibitors, can slow the disease but often cause severe side effects and drug resistance. In this study, we tested the effects of two kinase inhibitors, Sorafenib and Lapatinib, alone and in combination with Bevacizumab, an antibody that blocks blood vessel growth in tumors. We found that Sorafenib and Lapatinib were effective against medullary thyroid cancer cells, and when combined with Bevacizumab their effects were much stronger, even at lower doses. These findings suggest that combination therapy may be a promising way to improve treatment while reducing side effects, and they could guide future research into better strategies for patients with this challenging cancer.

**Abstract:**

**Background:** Medullary thyroid carcinoma is a rare neuroendocrine tumor with limited therapeutic options, as current kinase inhibitors are often associated with significant toxicity and drug resistance. This study aimed to explore novel treatment strategies by testing targeted agents alone and in combination. **Methods:** Human medullary thyroid carcinoma TT cells with RET mutations were treated with Sorafenib, Lapatinib, and Bevacizumab. Cell proliferation was monitored in real time using the xCELLigence system, and apoptosis was assessed by flow cytometry. **Results:** Sorafenib and Lapatinib each showed strong, dose-dependent cytotoxic effects, with Lapatinib demonstrating the greatest potency. Bevacizumab alone exhibited minimal cytotoxic activity, but when combined with Sorafenib or Lapatinib it significantly enhanced their effects, even at concentrations that were only partially effective individually. The Lapatinib–Bevacizumab combination produced the most potent inhibition of cell viability, comparable to high-dose monotherapy. **Conclusions:** These findings suggest that combining kinase inhibitors with Bevacizumab may enhance antitumor activity, allow the use of lower drug doses, and overcome resistance, representing a promising therapeutic strategy for medullary thyroid carcinoma that warrants further investigation in clinical settings.

## 1. Introduction

Thyroid cancer is one of the most common cancers worldwide, affecting people in both developing and developed countries, with an incidence of 600,000 new cases diagnosed annually [1]. It is the most prevalent endocrine malignancy globally, with approximately 586,202 cases reported in 2020, making it the tenth most common cancer worldwide [2].

Medullary thyroid carcinoma (MTC) is a neuroendocrine tumor of the parafollicular C cells of the thyroid [3]. Only 1–2% of all thyroid cancers are MTC [2]. Despite the low incidence, there is a mortality burden of up to 15% of all thyroid cancer-related deaths due to MTC [4]. It is noteworthy that the incidence of thyroid cancer has seen a significant rise, with a fourfold increase in females and a threefold increase in males over the past two decades [5]. Although the overall incidence of thyroid cancer has increased, the survival rates decrease with the advancement of the cancer stage [6]. This trend underscores the critical need for early diagnosis and effective therapeutic strategies to mitigate the impact of disease progression [7,8]. This is particularly true for medullary thyroid carcinoma, which, despite its rarity, contributes disproportionately to thyroid cancer mortality, with 8.6% of thyroid cancer-related deaths attributed to it [9]. MTC accounts for 3–10% of all thyroid cancers, typically presenting sporadically in individuals between 40 and 60 years of age, though hereditary forms linked to *RET* germline mutations can manifest earlier and account for 20–30% of cases [10].

Although surgical approaches are the first line of treatment for MTC, targeted therapies are a management option for patients with progressive or symptomatic disease with locoregional or metastatic MTC [11]. Targeted therapies have helped supplant cytotoxic chemotherapy, which has low efficacy against disease progression. However, there is still the problem of toxic side effects and frequent development of tumor resistance when using the current treatment regimen [12]. These problems indicate that there is still a need to develop an effective treatment for MTC [13]. In MTC mutations in the RET protooncogene lead to the overexpression of the receptor tyrosine kinase, resulting in increased activity in the cellular pathways responsible for proliferation, angiogenesis, and apoptosis. This process is responsible for tumorigenesis in all described cases of hereditary MTC and 40–50% of sporadic MTC cases [12].

Thyroid cancers are vascular tumors that overexpress vascular endothelial growth factor (VEGF) [13]. It is not surprising that Sorafenib, an orally active multikinase inhibitor of the tyrosine kinases including BRAF, rearranged during transfection (RET), FLT-3, KIT, VEGFR-1, VEGFR-2, VEGFR-3, and platelet-derived growth factor receptor-β [14], has been investigated extensively for this indication and finally proved its efficacy and have led to the design of the Phase III DECISION trial [15]. The DECISION trial showed that Sorafenib is a viable therapeutic option in advanced thyroid cancers. Beyond sorafenib, other multikinase inhibitors like vandetanib and cabozantinib have also gained regulatory approval for treating advanced medullary thyroid carcinoma, primarily targeting pathways involved in tumor growth and angiogenesis [16,17].

Although Sorafenib has promising results in treating MTC, one big problem with tyrosine kinase inhibitors is tolerability [16].

To address this, we investigated the anticancer effects of Sorafenib on MTC, both as a standalone treatment and in combination with the VEGF inhibitor Bevacizumab, with the goal of reducing the dosage and minimizing the side effects of tyrosine kinase inhibitors. We further explored the potential of another kinase inhibitor, Lapatinib, as a candidate for combination with Bevacizumab.

This comprehensive investigation aims to identify novel synergistic therapeutic strategies to overcome limitations associated with current MTC treatments, particularly resistance mechanisms and adverse event profiles [18].

## 2. Materials and Methods

### 2.1. Drugs and Cell Culture

Sorafenib (500 nM), Lapatinib (500 nM), and Bevacizumab (500 nM) were prepared as stock solutions dissolved in double-distilled sterile water. Before use, the stock solution was re-diluted to the desired concentrations in double distilled sterile water. Our study used the human thyroid medullary cancer cell line TT (CRL-1803™) with C634R RET mutation-positive epithelial-derived adhesion from the American Type Cell Collection (ATCC). Cells were grown in 25 cm^2^ flasks in Dulbecco’s Modified Eagle’s Medium (DMEM; Sigma-Aldrich Chemie, Taufkirchen, Germany) containing 10% FBS (Biochrom KG, Berlin, Germany) and 1% penicillin–streptomycin (Biochrom KG, Berlin, Germany) at 37 °C in a humidified atmosphere of 5% carbon dioxide in the air. This study was approved by Sivas Cumhuriyet University Non-Invasive Clinical Research Ethics Committee with the meeting decision dated 24 July 2014. It was studied in the laboratories of Sivas Cumhuriyet University Faculty of Medicine, Department of Pharmacology, and Faculty of Medicine Research Center (CUTFAM). In addition, the study was supported by Sivas Cumhuriyet University Scientific Research Projects Center with project number T-610.

### 2.2. xCELLigence System

The real-time cell analysis system (xCELLigence system) consists of four main components: RTCA analyzer, RTCA DP station, RTCA computer with integrated software, and E-plate 16. It forms the basis of the E-plate16 xCELLigence system used to perform cell-based experiments on the RTCA DP instrument. The gold plates, 80% of which are covered with circular electrodes on the ground of the plates, allow the application of the test procedure separately in each well and the evaluation of the results separately. The electronic impedances of the sensor electrodes are measured, allowing for the detection and monitoring of the physiological changes in the cells on the electrodes. This system is based on recording the electronic impedance of e-plates, including gold microelectrodes. The impedance of electron flow caused by adherent cells is reported using a unitless parameter called Cell Index.

The in vitro cytotoxic activities of Sorafenib, Lapatinib, and Bevacizumab used in our study were evaluated with a real-time cell analysis system. The xCELLigence system was used according to the manufacturer’s instructions (Roche Applied Science and ACEA Biosciences). First, a basal reading was taken on the instrument by adding 100 µL of the cell-free medium mixture to each well. Then, TT cells were trypsinized and counted by a hemocytometer. Cells were seeded into 16-well E-plates at 1 × 10^4^ cells in 100 µL in triplicate and incubated for 24 h in cell culture conditions. After the cells entered the rapid growth phase, the agents to be evaluated for their cytotoxic activity were applied to the wells determined as the experimental group at increasing concentrations in a volume of 10 µL. Cell index-cell content (CI) values were recorded every 15 min for 24 h. Wells containing DMEM were used as negative controls for baseline measurements of resistivity. Cytotoxicity was assessed by comparing the viability of cells treated with Sorafenib, Lapatinib, and Bevacizumab with the viability of untreated control cells using xCELLigence RTCA software (V2.8.1). Calculation of IC50 was performed by nonlinear regression analysis (Sigmoidal dose–response [variable slope]) [19].

### 2.3. Apoptosis Assay

Apoptosis was assigned by flow cytometry analysis of phosphatidylserine externalization. The Muse^®^ Annexin V & Dead Cell Kit was used on the Muse^®^ Cell Analyzer (Merck Millipore, Burlington, MA, USA) for the quantitative determination of apoptotic and non-apoptotic cells according to the manufacturer’s instructions. Briefly, the TT cells were seeded at a density of 6 × 105 cells/well in a six-well plate. The cells were incubated with Bevacizumab, Sorafenib, Lapatinib, Sorafenib + Bevacizumab, Lapatinib + Bevacizummab at concentrations of 50% inhibition of cell growth (IC50) 11.75 μM, for 24 h. After handling with the compound, the cells were gathered, resuspended with PBS including 1% FBS and maintained with the Muse^®^ Apoptosis Assay kit reagent for 20 min at 20–22 °C in the dark. After incubation, the stained cells were analyzed using a Muse^®^ Cell Analyzer (Merck Millipore, Burlington, MA, USA) to quantify the percentages of viable, early apoptotic, late apoptotic, and dead cells [20].

### 2.4. Statistical Analysis

SPSS 25.0 (SPSS Inc., Chicago, IL, USA) was used for the data analysis. All data were expressed as the mean ± standard error of the mean, with at least three independent experiments. The difference analysis of variance between groups (ANOVA test) followed by the post hoc TUKEY test (when required) was used. *p* < 0.05 was considered statistically significant.

## 3. Results

### 3.1. Protocol for Determining Optimum Cell Count

**Figure 1:**
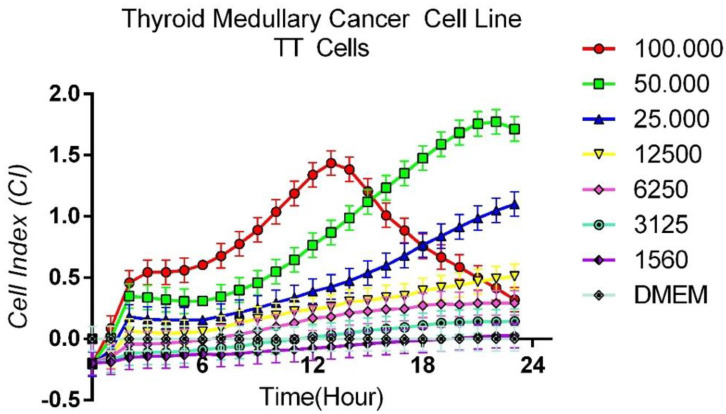
Time-dependent growth patterns of TT cells.

In order to determine the initial cell number to be used in cytotoxicity experiments, cells were prepared in various dilutions and applied to E-plates. Growth patterns were evaluated by seeding cells at 100,000, 50,000, 25,000, 12,500, 6250, 3125 and 1560 cells per well (Figure 1). It was determined that the cells showed a concentration-dependent proliferation. When the growth profiles of the cells were evaluated, it was decided that 50,000 cells per well were the most appropriate number for further use in the study.

### 3.2. Cytotoxic Effects of Sorafenib on TT Cells

**Figure 2:**
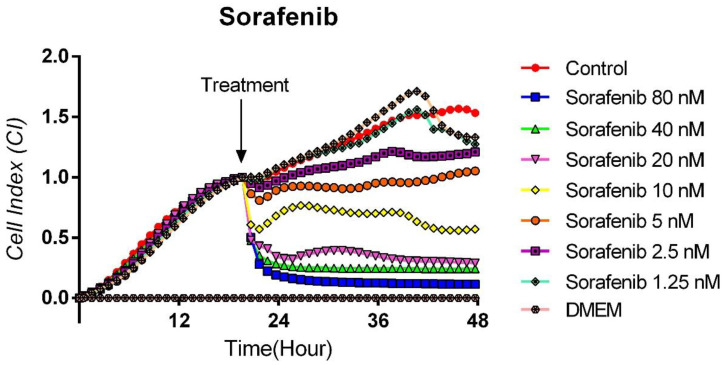
Sorafenib proliferation analysis on the TT cells. Cell proliferation was measured in real time for 48 h in the studied concentration range of Sorafenib (1.25–80 nM). Cell index (CI), which is the change in the electrical impedance of the cell increase, is graphed against time. (*p* < 0.05).

The antitumoral effect of 80, 40, 20, 10, 5, 2.5, and 1.25 nM concentrations of Sorafenib, a tyrosine kinase inhibitor, on human medullary thyroid cancer cell line TT, was evaluated with the real-time cell analysis system (xCELLigence System). The TT cells showed an exponential proliferation profile with a cell index close to 1 for 24 h. Sorafenib was administered at the end of the 24th hour, and cytotoxicity was monitored by real-time measurements until the 48th hour. Figure 2 shows significant inhibition of proliferation was observed in cells treated with Sorafenib at concentrations of 80, 40, and 20 nM such that the cell index decreased to 0.1. It was determined that 10 nM Sorafenib reduced cell viability by 50%. However, it was observed that other concentrations had no effect on the proliferation of TT cells and the growth curves were parallel to the control group. The IC50 for Sorafenib was calculated as 10.2 × 10^−9^ M, while the curve fit value was calculated as Square R = 0.999 (Figure 2).

### 3.3. Cytotoxic Effects of Lapatinib on TT Cells

**Figure 3:**
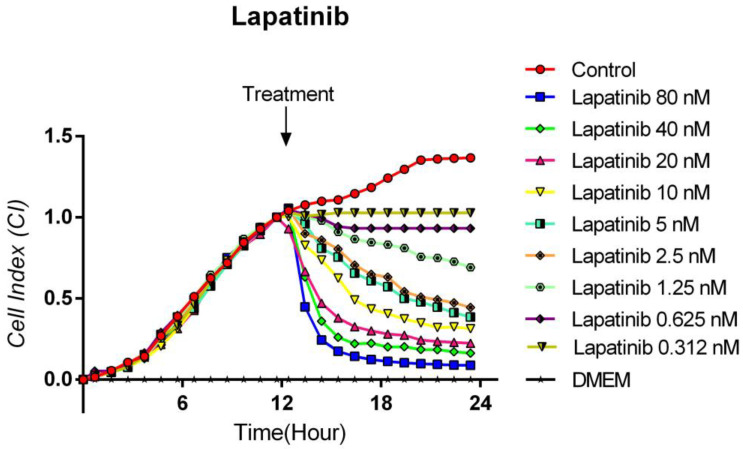
Lapatinib proliferation analysis on the TT cells. Cell proliferation was measured in real time for 48 h in the studied concentration range of Lapatinib (0.312–80 nM). Cell index (CI), which is the change in the electrical impedance of the cell increase, is graphed against time. (*p* < 0.05).

The antitumoral effect of increasing concentrations of a tyrosine kinase inhibitor Lapatinib (80, 40, 20, 10, 5, 2.5, 1.25, 0.625, and 0.312 nM) was evaluated on human medullary thyroid cancer cell line TT, with the real-time cell analysis system (xCELLigence System). The TT cells showed an exponential proliferation profile for 12 h. Lapatinib was administered at the end of the 12th hour, and cytotoxicity was monitored by real-time measurements until the 24th hour. It was determined that Lapatinib showed a strong cytotoxic effect dependent on concentration and time. Figure 3 shows significant inhibition of proliferation in cells treated with Lapatinib at concentrations of 80, 40, and 20 nM, such that the cell index decreased to 0.1. Even at the lowest concentration of 0.312 nM, Lapatinib showed a statistically significant potent cytotoxic effect when compared to the control. It was observed that the remaining concentrations were homogeneously distributed. The IC50 for Lapatinib was calculated as 1.2 × 10^−9^ M, while the curve fit value was calculated as Square R = 0.927 (Figure 3).

### 3.4. Cytotoxic Effects of Bevacizumab on TT Cells

**Figure 4:**
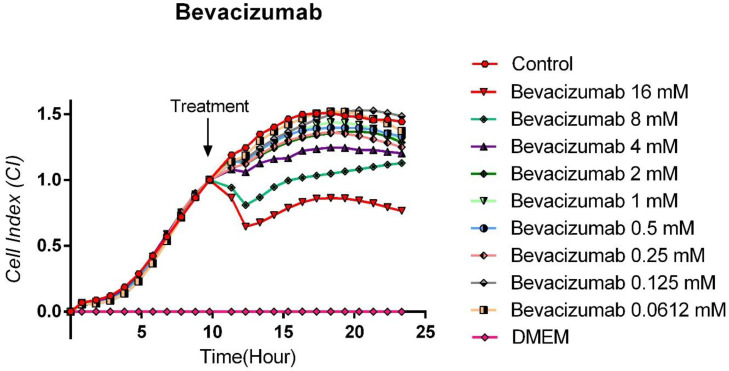
Bevacizumab proliferation analysis on the TT cells. Cell proliferation was measured in real time for 48 h in the studied concentration range of Bevacizumab (0.0612–16 mM). Cell index (CI), which is the change in the electrical impedance of the cell increase, is graphed against time. (*p* < 0.05).

The antitumoral effect of 16, 8, 4, 2, 1, 0.5, 0.25, 0.125, and 0.0612 mM concentrations of Bevacizumab, a specific VEGF antibody, on human medullary thyroid cancer cell line TT, was evaluated with the real-time cell analysis system (xCELLigence System). It was observed that Bevacizumab did not show a statistically significant cytotoxic effect on medullary thyroid cancer cells (*p* > 0.05). It was observed that only a very high concentration of 16 mM inhibited the proliferation in 47.5% of the cells, and this effect was tried to be balanced with an immediately opposite effect (rebound proliferative effect). In parallel, it was observed that 8 mM Bevacizumab concentration also caused a partial cytotoxic effect, but because this effect was very weak, this cytotoxic effect was balanced with the increase in proliferation rates of cells due to reflex transcription factor activation. The other concentrations were observed to have no cytotoxic effects, and the proliferation curves were parallel to the control group cells. The IC50 for Bevacizumab was calculated as 18.3 × 10^−3^ M, while the curve “fit value” was calculated as Square R = 0.914 (Figure 4).

### 3.5. The Comparison of the Cytotoxic Effects of Sorafenib, Lapatinib, and Bevacizumab

**Figure 5:**
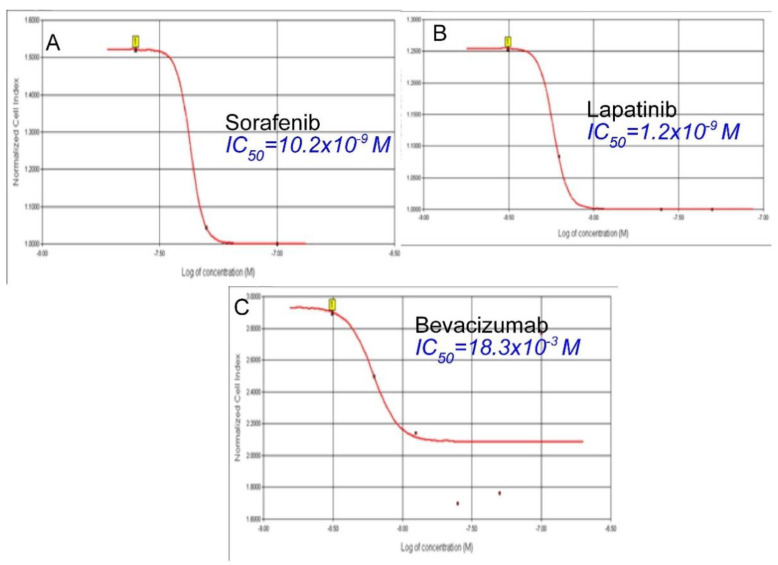
The determination of the IC50s of (**A**) Sorafenib, (**B**) Lapatinib, and (**C**) Bevacizumab.

**Figure 6:**
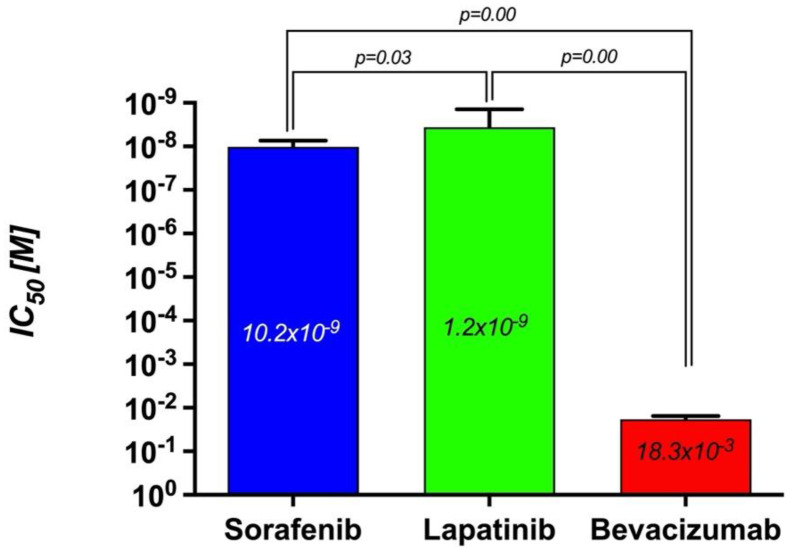
The comparison of the IC50 of Sorafenib, Lapatinib, and Bevacizumab.

The cytotoxic effects of the targeted therapy agents used in this study alone on TT cells were compared using IC50 values. The tyrosine kinase inhibitor Lapatinib had the most potent cytotoxic effect (Figure 5B), while the monoclonal VEGF antibody Bevacizumab had the lowest cytotoxic effect (Figure 5C). It was determined that the IC50 value of Sorafenib was higher than Lapatinib, and the difference was statistically significant (*p* = 0.000). The order of agents according to their IC50 values is Lapatinib < Sorafenib < Bevacizumab (Figure 6). Considering that the gravimetric effect of the agent with a lower IC50 value is higher, the order of the agents in terms of their cytotoxic effect on medullary thyroid cancer cells is Lapatinib > Sorafenib > Bevacizumab.

### 3.6. The Proliferation Analysis of the Sorafenib + Bevacizumab Combination on TT Cells

**Figure 7:**
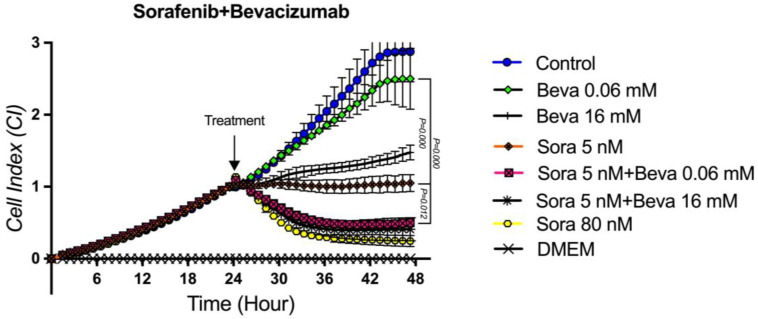
Sorafenib–Bevacizumab combination proliferation analysis on the TT cells. Cell proliferation was measured in real time for 48 h in the studied concentration range of Sorafenib–Bevacizumab. Cell index (CI), which is the change in the electrical impedance of the cell increase, is graphed against time. (*p* < 0.05).

In order to determine the cytotoxic effect of the combined applications of Sorafenib (5 nM) and Bevacizumab (16 mM), which were found to have antitumoral activities on TT medullary thyroid cancer cell proliferation alone, the concentrations at which Sorafenib and Bevacizumab showed partial activity were applied in combination. In addition, in order to determine whether there is an additive drug interaction between Sorafenib and Bevacizumab, the cytotoxic activity on TT cells was evaluated by combining the concentration where Sorafenib was partially effective (5 nM) and the concentration at which Bevacizumab was not effective (0.06 mM). When the concentrations of Sorafenib and Bevacizumab showing partial cytotoxic activity (5 nM and 16 mM) were applied in combination, it was observed that the cytotoxic effect of the combination was statistically significantly stronger when compared to the cytotoxic activity of the agents alone (*p* = 0.012, *p* = 0.09, respectively). This result shows that the co-administration of the agents significantly increases the cytotoxic activity of each other. There was no statistical difference between the cytotoxic effect of this combination and the cytotoxic effect of 80 nM Sorafenib alone (*p* = 0.874). More interestingly, when the concentration of Sorafenib (5 nM) showing partial cytotoxic activity was combined with the concentration of Bevacizumab (0.06 mM) at no cytotoxic effect, it was observed that the cytotoxic effect of the combination was statistically significantly stronger than the concentrations at which each of the agents was administered alone (5 nM and 0.06 mM) (*p* = 0.000 and *p* = 0.012, respectively).It was observed that there was no statistically significant difference between the cytotoxic potency of this combination and the combined administration of Sorafenib 80 nM alone and Sorafenib 5 nM + Bevacizumab 16 mM combined (*p* = 0.932 and *p* = 896), respectively. This suggests that the partial cytotoxic concentration of Sorafenib at a concentration at which Bevacizumab alone is ineffective additively enhances its effect, contributing to a 16-fold more substantial effect (Figure 7).

### 3.7. The Proliferation Analysis of the Lapatinib + Bevacizumab Combination on TT Cells

**Figure 8:**
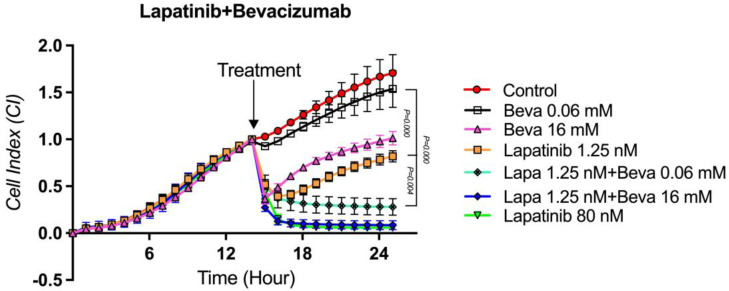
Lapatinib + Bevacizumab combination proliferation analysis on the TT cells. Cell proliferation was measured in real time for 48 h in the studied concentration range of Lapatinib + Bevacizumab. Cell index (CI), which is the change in the electrical impedance of the cell increase, is graphed against time. (*p* < 0.05).

In order to determine the cytotoxic effect of the combined applications of Lapatinib and Bevacizumab agents, which were found to have antitumoral activities on TT medullary thyroid cancer cell proliferation alone, the concentration at which Lapatinib (1.25 nM) had partial activity and the concentration at which Bevacizumab (16 mM) had partial activity were applied in combination. In addition, in order to determine whether there is an additive drug interaction between Lapatinib and Bevacizumab, the cytotoxic activity on TT cells was evaluated by combining the concentration at which Lapatinib (1.25 nM) had partial activity and the concentration at which Bevacizumab (0.06 mM) had no activity. When the concentrations of Lapatinib and Bevacizumab showing partial cytotoxic activity (1.25 nM and 16 mM) were applied together, it was observed that the cytotoxic effect of the combination was statistically significantly stronger than the cytotoxic effect of the agents alone (*p* = 0.008 and *p* = 0.05, respectively). This result shows that the co-administration of the agents significantly increases the cytotoxic effect of each other. There was no statistical difference between the cytotoxic effect of this combination and the cytotoxic effect of 80 nM Lapatinib alone (*p* = 0.914). More interestingly, when the partial cytotoxic concentration of Lapatinib (1.25 nM) was combined with the concentration of Bevacizumab (0.06 mM) at no cytotoxic effect, it was observed that the cytotoxic effect of the combination was statistically significantly stronger than the concentrations at which each of the agents was administered alone (1.25 nM and 0.06 mM) (*p* = 0.000 and *p* = 0.004, respectively). It was observed that there was no statistically significant difference between the cytotoxic effect of Lapatinib 80 nM alone and the combined application of Lapatinib 1.25 nM + Bevacizumab 16 mM (*p* = 0.946 and *p* = 0.996, respectively). This suggests that Bevacizumab at an ineffective concentration alone contributes to a 64-fold stronger effect by additively increasing the effect of Lapatinib at a concentration with a partial cytotoxic effect (Figure 8). When the graphs of the cytotoxic effects of the combination applications are compared, it is predicted that the Lapatinib + Bevacizumab combination has a stronger cytotoxic effect than the Sorafenib + Bevacizumab combination.

### 3.8. Evaluation of Apoptosis by Flow Cytometry

To elucidate the mechanism of cell death induced by the tested agents, apoptosis was assessed using Annexin V staining and flow cytometry analysis in TT cells following 24-h treatment with the IC_50_ concentration of each drug, both alone and in combination.

**Figure 9:**
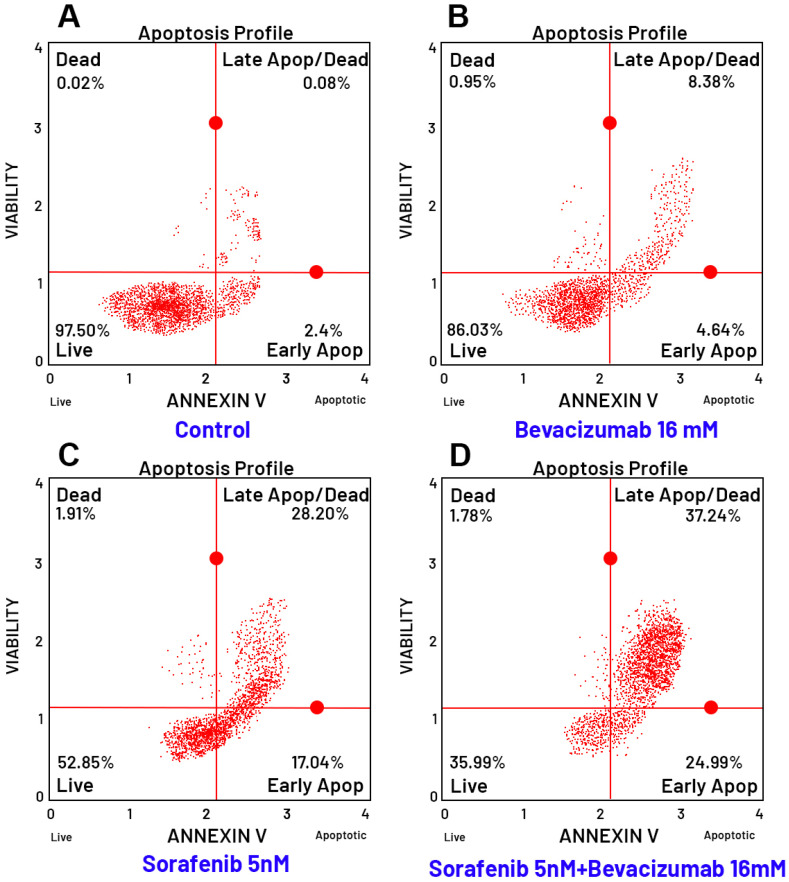
Apoptosis analysis of TT cells treated with the Sorafenib and Bevacizumab combination. Flow cytometry histograms (**A**–**D**) showing Annexin V staining in TT cells after 24-h treatment under the following conditions: (**A**) Untreated control, (**B**) Sorafenib (IC_50_ concentration), (**C**) Bevacizumab (IC_50_ concentration), and (**D**) Sorafenib + Bevacizumab combination. The combination treatment resulted in a marked increase in the percentage of apoptotic (Annexin V–positive) cells compared to the control and single-agent treatments. Red lines indicate the quadrant gates automatically generated by the flow cytometry software to distinguish viable, early apoptotic, late apoptotic, and dead cell populations. Note: The control and Bevacizumab panels are derived from the same experimental dataset used consistently across Figure 9 and Figure 10. Both analyses were performed simultaneously in parallel cultures under identical experimental conditions to ensure comparability between Sorafenib–Bevacizumab and Lapatinib–Bevacizumab combinations.

Quantitative analysis revealed that Sorafenib alone significantly induced apoptosis compared to the control group (Figure 9). While the untreated control group exhibited a high viability rate (97.50% live cells), Sorafenib treatment drastically reduced the viable cell population to 52.85%, with a corresponding increase in early apoptosis (17.04%) and late apoptosis/necrosis (28.20%). Bevacizumab monotherapy showed a modest effect, reducing viability to 86.03%. Crucially, the combination of Sorafenib with Bevacizumab demonstrated a synergistic enhancement of the pro-apoptotic effect. The viable cell population was further reduced to 35.99%, while early and late apoptotic cells increased to 24.99% and 37.24%, respectively. This indicates that the addition of Bevacizumab significantly potentiates Sorafenib-induced cell death.

**Figure 10:**
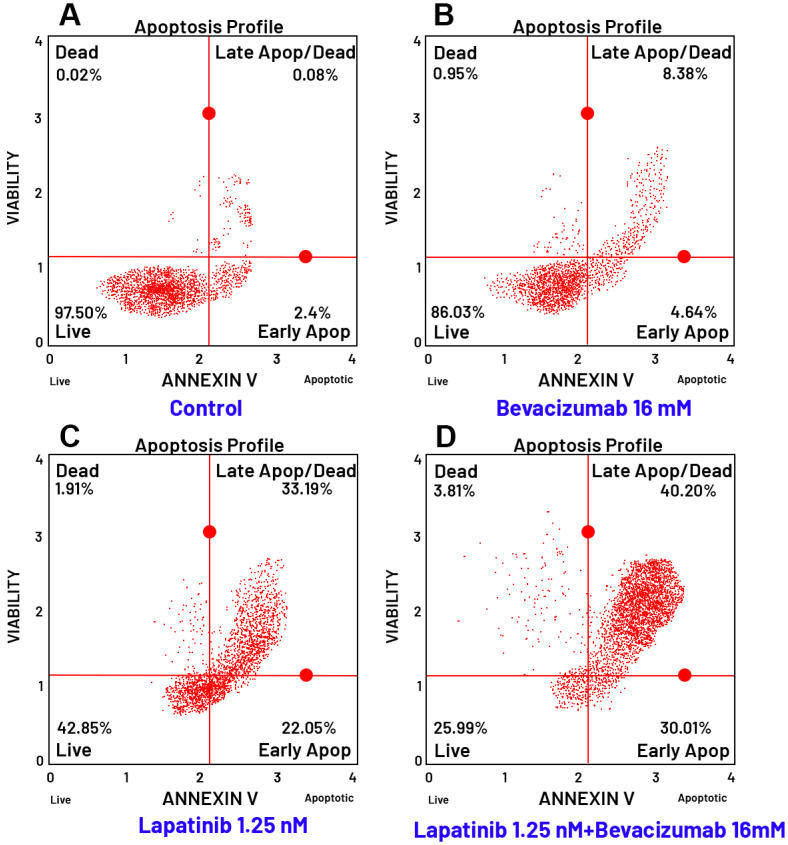
Apoptosis analysis of TT cells treated with the Lapatinib and Bevacizumab combination. Flow cytometry histograms (**A**–**D**) showing Annexin V staining in TT cells after 24-h treatment under the following conditions: (**A**) Untreated control, (**B**) Lapatinib (IC_50_ concentration), (**C**) Bevacizumab (IC_50_ concentration), and (**D**) Lapatinib + Bevacizumab combination. The Lapatinib–Bevacizumab combination demonstrated a potent pro-apoptotic effect, significantly increasing the Annexin V–positive cell population compared to both control and monotherapy groups. Red lines indicate the quadrant gates automatically generated by the flow cytometry software to distinguish viable, early apoptotic, late apoptotic, and dead cell populations. Note: The control and Bevacizumab panels correspond to the same dataset presented in Figure 9, reused here for consistency and direct comparison between the two combination analyses, as both were derived from the same experimental series performed in triplicate.

A similar potentiation was observed with the Lapatinib and Bevacizumab combi-nation (Figure 10). Lapatinib alone exhibited a strong cytotoxic profile, reducing viability to 42.85% and inducing 22.05% early apoptosis and 33.19% late apoptosis/necrosis. The combination with Bevacizumab resulted in the most profound reduction in cell viability among all treatments, with only 25.99% of cells remaining viable. Furthermore, this combination yielded the highest percentages of early apoptotic (30.01%) and late apoptotic/necrotic (40.20%) cells, underscoring a powerful synergistic interaction between Lapatinib and Bevacizumab in triggering programmed cell death.

**Figure 11:**
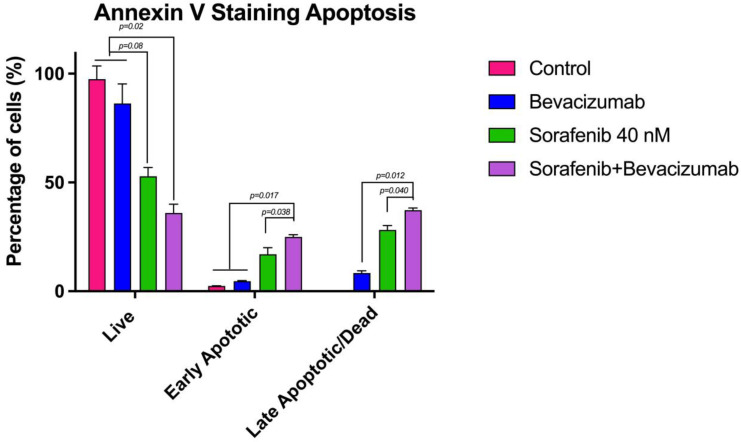
Quantitative analysis of apoptosis induced by the Sorafenib and Bevacizumab combination. Bar graph quantitatively comparing the percentages of viable and apoptotic TT cells after 24-h treatment with the Sorafenib–Bevacizumab combination versus the control, as assessed by Annexin V staining. Data are presented as mean ± SEM. The combination treatment significantly reduced cell viability and increased apoptosis.

**Figure 12:**
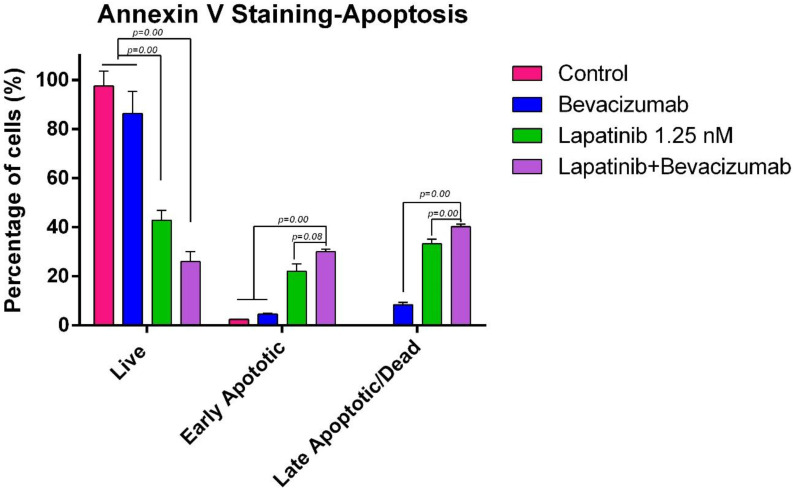
Quantitative analysis of apoptosis induced by the Lapatinib and Bevacizumab combination. Bar graph quantitatively comparing the percentages of viable and apoptotic TT cells after 24-h treatment with the Lapatinib–Bevacizumab combination versus the control, as assessed by Annexin V staining. Data are presented as mean ± SEM. The combination treatment resulted in a dramatic reduction in viable cells and a corresponding increase in apoptosis.

The apoptotic effects of the combinations were further confirmed by quantitative bar graphs (Figure 11 and Figure 12), which clearly illustrate the significant shift from viable to apoptotic cell populations upon combination treatment compared to single-agent therapies. Collectively, these apoptosis data strongly correlate with the cytotoxicity results, confirming that the enhanced anti-proliferative effects of the drug combinations are mediated through the activation of apoptotic pathways. The Lapatinib–Bevacizumab combination emerged as the most effective regimen in inducing apoptosis in MTC cells.

## 4. Discussion

Thyroid cancer is the most prevalent endocrine malignancy, which accounts for approximately 4% of all malignancies. Its overall incidence has increased over the years [21]. Although surgery has been the most effective treatment to reduce the tumor burden of ATC [22], the prognosis remains very poor, with a mean survival time after diagnosis of 6 months [23]. Furthermore, the survival rate of patients with ATC has not improved over the past 20 years. Tyrosine kinase inhibitors (TKIs) are small molecules that target intracellular signaling pathways and are strongly preferred in the medical treatment of MTC. Although promising, these drugs show only a modest effect on advanced thyroid cancer and exhibit significant toxicity [24]. Although promising, these drugs show only a modest effect on advanced thyroid cancer, while they exhibit significant toxicity. Avoiding severe secondary effects while optimizing the efficiency of conventional chemotherapy is still a huge challenge in cancer treatment today. That is why its combination with new drugs is now so popular to minimize adverse reactions by reducing the doses of toxic molecules and to play on synergism to achieve the efficiency needed against tumors [25]. In the present study, we first investigated the cytotoxic effects of Sorafenib, Lapatinib, and Bevacizumab alone to determine the inhibitory concentration fifty (IC50) values for further phases of the study. We showed that Sorafenib had time and concentration-dependent potent cytotoxicity on TT MTC cells, which is consistent with the literature. Carlomogno et al. [26] investigated the cytotoxic effects of BAY-43-9006 (Sorafenib) on oncogenic RET mutant TT cells in 2006 for the first time. They showed its antiproliferative effect on TT cells when it was only a code-name molecule. Even though the IC50 value is not calculated, the concentrations used in the study are like ours. Then, Lin et al. [27] and Koh et al. [28] investigate the cytotoxic effects of Sorafenib on TT TMC cell lines through autophagy and MEK inhibition, respectively.

Both studies demonstrated a concentration-dependent solid antiproliferative effect similar to our research. Yet we proposed induction of apoptosis as one of the major mechanisms of Sorafenib, which differs from the other studies. Finally, O’Brien et al. [29]. acknowledge the fact that although small molecules TKIs are very good at killing thyroid cancer cells, resistance is a big problem in maintaining the treatment. So, they conducted a study where Focal adhesion kinase (FAK) inhibition is combined with some conventional TKIs, including Cabozantinib, Sorafenib, and Pazopanib, in order to overcome the resistance. When administrated alone, they showed FAK inhibitor Y15 has an antiproliferative effect on K1 and TT cells. But they only tried the combination of Y15 and Sorafenib on K1 cells since FAK activation is much stronger in the papillary type of thyroid cancers. This combination approach is then applied in clinical trials as well. Massicotte et al. combined Sorafenib with Sunitinib, whereas Hong et al. [30] combined Sorafenib with Tififarnib. Both studies showed an increase in cytotoxicity when Sorafenib was combined with another TKI.

We believe combining a TKI with other TKIs may increase cytotoxicity. Nonetheless, since both drugs affect the same downstream pathway, choosing a combination drug with another mechanism of action may be wiser. So, we have chosen Bevacizumab, a humanized monoclonal antibody that targets VEGF-A, as a combination agent. Bevacizumab has been shown to be effective in colorectal cancer, non-small-cell lung cancer, renal cell carcinoma, ovarian carcinoma, cervical cancer, and glioblastoma multiforme as a neoadjuvant agent to combine it with the conventional treatment agent of the respected cancer type [31,32]. Similarly to all these studies in the literature, the combination of Sorafenib with Bevacizumab in the present study decreased the cell viability of TT cells and increased apoptosis significantly.

Moreover, the Lapatinib + Bevacizumbam combination also increased the cytotoxicity, and it was much more potent when compared to the combination with Bevacizumab.

Since Vandetanib and Cabozantinib are FDA-approved major drugs in the treatment of TMC and Sorafenib was recently approved for patients with RAI-resistant distant metastases and are part of the National Comprehensive Cancer Network and the American Thyroid Association guidelines [33]. Yet Vandetanib and Cabozantinib are not available in every country. Sorafenib has been used in Turkey with special permission from the Ministry of Health for this off-label indication in treating these patients [34]. This increases the importance of Sorafenib and makes it a helpful tool.

## 5. Conclusions

This study demonstrated the significant efficacy of Sorafenib in treating MTC, with combination therapies notably enhancing its potency. Sorafenib and Lapatinib, in combination with Bevacizumab, emerge as promising options for MTC treatment, offering potential for increased cell toxicity, reduced side effects, and improved management of drug resistance. The findings suggest that strategic combinations of targeted therapies, such as pairing tyrosine kinase inhibitors with anti-angiogenic agents like Bevacizumab, could represent a more effective and tolerable approach to managing advanced thyroid cancer, including MTC. Further clinical investigation is warranted to fully elucidate the therapeutic benefits and optimal dosing strategies for these combinations in a broader patient population.

## Data Availability

The data supporting this study are not publicly available due to privacy and ethical restrictions. In compliance with the strict data protection policies of the Ministry of Health of the Republic of Türkiye and the national Personal Data Protection Law (Law No. 6698, KVKK), raw or individual-level data cannot be shared publicly. Limited, anonymized data may be provided by the corresponding author upon reasonable request and with appropriate institutional and ethical approval.
